# What is the evidence base for diagnosing hypertension and for subsequent blood pressure treatment targets in the prevention of cardiovascular disease?

**DOI:** 10.1186/s12916-015-0502-5

**Published:** 2015-10-12

**Authors:** Claire L. Schwartz, Richard J. McManus

**Affiliations:** Nuffield Department of Primary Care Health Sciences, National Institute for Health Research (NIHR) National School for Primary Care Research, University of Oxford, Oxford, OX2 6GG UK

**Keywords:** ABPM, Blood pressure, Hypertension diagnosis, Hypertension guidelines, Self-monitoring, Thresholds, Treatment targets

## Abstract

Diagnosing and treating hypertension plays an important role in minimising the risk of cardiovascular disease and stroke. Early and accurate diagnosis of hypertension, as well as regular monitoring, is essential to meet treatment targets. In this article, current recommendations for the screening and diagnosis of hypertension are reviewed. The evidence for treatment targets specified in contemporary guidelines is evaluated and recommendations from the USA, Canada, Europe and the UK are compared. Finally, consideration is given as to how diagnosis and management of hypertension might develop in the future.

## Background

Hypertension affects around 40 % of the worldwide population aged over 25 years, and is estimated to be implicated in approximately half of the deaths from stroke or cardiovascular disease [1]. Early and accurate diagnosis is essential in order to appropriately manage hypertension and reduce these risks, but national and international surveys suggest that many people continue to have unrecognised or untreated hypertension, with variation between countries [2, 3]. Here, the evidence on screening and diagnosis of hypertension is reviewed and consideration given as to optimum treatment targets for preventing cardiovascular disease in patients.

### What is the best way to diagnose hypertension?

Traditionally, hypertension has been diagnosed using clinic blood pressure (BP) measurements, typically taking several readings on several occasions and then treating those whose mean BP is consistently above the diagnostic threshold [4–6]. However, since the advent of both ambulatory blood pressure monitoring (ABPM) and self-measured blood pressure (SMBP), it has been recognised that measurements outside of a clinic environment are better correlated with long-term outcomes [7–11].

ABPM is widely regarded as the “gold standard” for BP measurement. For example, both the Dublin outcome study, involving 5,292 patients, and a meta-analysis of 7,030 individuals from the International Database of Ambulatory Blood Pressure in relation to Cardiovascular Outcome (IDACO) database, found that systolic and diastolic ABPM measurements significantly and independently predicted cardiovascular outcomes over and above clinic BP [12, 13]. Furthermore, the Ohasama study (1,464 subjects, general population, 6- to 9-year follow-up), found that mean ambulatory pressure, especially mean daytime, was linearly related to stroke risk and was a stronger predictor than clinic BP [14]. Other measures of blood pressure, particularly nighttime pressure, have been suggested as superior but may not add much more in terms of prognosis than the 24-hour mean itself [15]. ABPM also allows identification of masked and white coat hypertension and is reproducible [16–19]. Masked hypertension carries significant cardiovascular risk [20], which is estimated to be similar to sustained hypertension [21, 22].

There is also strong evidence for the prognostic accuracy of SMBP over clinic BP. SMBP is a significantly stronger predictor of cardiovascular outcomes [11, 23, 24], stroke [25, 26] and target organ damage [24, 27] than clinic BP. A recent systematic review found that the association of SMBP with target organ damage was as strong as that from ABPM [28].

Very few studies have assessed SMBP, ABPM and clinic BP against each other for cardiovascular outcomes. Fagard et al. compared these three methods of BP measurement for predicting cardiovascular events over 10 years in 391 older patients [29]. The authors found that the prognostic value of self-monitoring and daytime ABPM were similar to office measurements in predicting cardiovascular outcomes, but the strongest independent predictor was nighttime ABPM.

In a systematic review, Hodgkinson et al. evaluated 20 studies that had made a hypertension diagnosis with clinic or home BP measurement compared to ABPM as reference standard [30]. Using a clinic diagnostic threshold of 140/90 mmHg and ambulatory and self-monitoring thresholds of 135/85 mmHg, the review found neither home nor clinic measurements to be sufficiently accurate compared to ABPM, although others have argued that BP differences for those with discordant ABPM and self-monitoring are small [31, 32]. Furthermore, only three SMBP studies were available at the time. Since then, a number of other studies have been reported, including Nasothimiou [31] and Nunan [33]. The former studied patients attending a hypertension clinic and recorded high diagnostic test performance for self- compared to ambulatory monitoring, whereas Nunan’s paper reported high sensitivity and only modest specificity, but utilised a community-based cohort with a lower prevalence of sustained hypertension (54 % versus 65 % in untreated subjects).

Current diagnostic thresholds for out-of-office measurement are based on work by Head et al., which compared 8,575 ABPM measurements to contemporaneous clinic readings taken by trained staff [34]. They found that the equivalent daytime mean ABPM for an office measurement of 140/90 mmHg was 4/3 mmHg less, which led to an ABPM target of 135/85 mmHg. However, work by Niiranen et al. suggested that the thresholds for SMBP are different and systolic thresholds in particular may be too high [35]. Based on a hypertensive population, Niiranen et al. found that thresholds of 130/85 and 145/90 mmHg for stages 1 and 2 hypertension better predicted outcomes for cardiovascular events over an 8-year follow-up.

A strong argument for using out-of-office measures arises from the concept of masked hypertension, where patients have a normal or controlled clinic BP but an elevated out-of-office measurement. Diagnosis of this condition is important because patients can be untreated or undertreated putting them at greater risk of cardiovascular disease [21]. Banegas et al. analysed data from the Spanish Society of Hypertension ABPM registry [36]. Out of more than 14,000 patients, with treated and (apparently) controlled clinic BP, 31.1 % had masked uncontrolled hypertension, with the most likely reason being poor control of nighttime BP. Both ABPM and SMBP offer a way to diagnose and manage masked hypertension, but only if performed routinely in those with normal clinic BP. Hence more work needs to be carried out to understand the best way to target patients appropriately and manage masked hypertension in the longer term.

The cost-effectiveness of routine out-of-office measurement in the diagnosis of hypertension was assessed in a modelling exercise undertaken as part of the development of the National Institute for Health and Care Excellence (NICE) hypertension guideline [37]. This showed that the additional costs of ABPM were more than offset by the increased precision in diagnosis, and hence it was more cost-effective than either office BP measurement or SMBP for all age and gender subgroups. Long-term costs (in terms of cardiovascular events and reduced need for follow-up) were reduced when using ABPM instead of office BP measurement. There are potential issues with ABPM, including availability and feasibility [5]. However, given the need for life-long treatment following a diagnosis of hypertension, these are arguably insufficient to prevent implementation – a fact underpinned by the recent Canadian recommendations [38].

New technologies may provide a useful interface between non-physician screening and primary care. Telemonitoring and electronic submission of readings are becoming more popular [39] and, with smartphones becoming ever more sophisticated, it seems likely that this will play a role in diagnosing and managing hypertension in the future. There is growing evidence that telemonitoring in combination with self-monitoring is more effective than usual care in reducing blood pressure [40] and is acceptable to patients [41]. However, it is not widely used in clinical practice. The barriers to using telemonitoring include the initial set-up costs and issues around reimbursement.

A recent randomised controlled trial (RCT) using telemonitoring in the UK found that the direct mean cost of systolic BP reduction was £25.56/mmHg per patient compared to usual care [42]. Even though the telemonitoring intervention significantly reduced BP over 6 months, unless longer-term outcomes (cardiovascular events) are considered, the additional costs of telemonitoring may not be considered affordable. There are also differences between healthcare and telemonitoring systems, which can lead to apparently contradictory results, with some found to be cost-effective [43–45] but others not [46].

Another barrier, particularly significant to the USA, is the need for uniform quality standards which drive reimbursement. There is a lack of acceptance that patient-reported data meets quality measures, which stipulate that controls, such as face-to-face visits and physiologic measurements, adhere to certain specifications, for example nurse-only BP checks [47]. In theory, telemonitoring could fulfil these criteria and the American Telehealth Association is working to address such barriers and start implementing telehealth in primary care [48]. As the evidence base for using telemonitoring in primary care continues to expand, it is likely that we will start to see guidelines and standards for telemonitoring in the UK and Europe as well.

### When should patients have their BP measured?

In addition to the method of screening for hypertension, the frequency of such screening is also important, but the evidence to underpin this is scarce. Piper et al. tried to confirm “the shortest interval in which clinically significant, diagnosed hypertension may develop” in their recent systematic review [49]. They found 43 studies had examined screening intervals and established the incidence of hypertension found in 1- to 5-year intervals. It was impossible to reach any firm conclusions due to heterogeneity in the study results, with mean incidence ranging from 2 % to 28 % over a 5-year period [49]. It may be more helpful to consider when to rescreen on the basis of current BP. Five studies reported on the incidence of hypertension over a 5-year period for three categories of BP: optimum, <120/80 mmHg; normal, 120–129/80–84 mmHg; and high-normal, 130–139/85–89 mmHg [50–54]. Meta-analysis showed increased incidence of hypertension with increasing baseline blood pressure, a six-fold increase between high-normal and optimum, and a very low rate in the latter case – of less than 10 % in a 5-year period [49]. The authors suggest that this highlights the need to identify subpopulations which may benefit from a more structured screening programme, including older people, overweight or obese patients, those with a high-normal BP and certain ethnic minority groups [49].

Whilst primary care remains the commonest setting for hypertension screening, there is growing evidence to suggest that community screening may help to reach greater numbers of patients. A comprehensive systematic review by Fleming et al. showed that screening has been undertaken in a range of locations, with pharmacies and mobile units the most successful settings assessed, albeit with a high level of heterogeneity [55]. However, only 16 % of studies reported a referral to primary care following screening, of which a new hypertension diagnosis was made in a median of 44 %, suggesting that a joined-up approach is fundamental to the impact of such screening.

Novel primary care settings may also prove successful, such as optometry [56]. In the USA, screening for medical conditions in dentistry has been assessed as potentially acceptable to both patients and physicians [57, 58]. More studies in this area are needed to establish how physician and non-physician screening could complement each other.

Recently, a group of primary care practices in the USA developed an algorithm for identifying patients at risk of hypertension from their electronic records [59]. The innovation was successful and has now been implemented in these practices. It is easy to see how this type of innovation could be used in calculating rescreening intervals for patients and also follow-up times after BP treatment intensification.

### Where and how are patients currently diagnosed?

Since 2011, in the UK, guidelines from NICE have recommended that a raised clinic BP reading (≥140/90 mmHg) in an undiagnosed patient should be followed by confirmatory ABPM, unless BP ≥180/110 mmHg [37]. Home BP measurement can be used as an alternative if ABPM is not available or tolerated.

In comparison, the European Society of Hypertension (ESH) practice guidelines (2013) recommend that office BP remains the “gold standard” for screening, diagnosis and management of hypertension [60]. However, for diagnostic purposes they recommend that ABPM or home BP is used in the case of suspected white coat hypertension, masked hypertension, grade I hypertension (≥140/90 mmHg) and high-normal BP, which can be interpreted as a broad recommendation for use.

The Canadian Hypertension Education Program (CHEP) have recently updated their recommendations for the diagnosis of hypertension to include the use of ABPM and home BP monitoring [61]. For any office BP measurement >140/90 mmHg but <180/110 mmHg, out-of-office measurement should be used to rule out white coat hypertension. ABPM readings should be taken every 20–30 minutes during the day and every 30–60 minutes at night. At least 20 daytime readings and 7 nighttime readings are required to obtain an accurate average reading. Home BP should be measured using validated monitors that have met the standards set by the Association for the Advancement of Medical Instrumentation (AAMI). Hypertension diagnoses using home monitoring should be based on duplicate measurements taken morning and evening for a 7-day period, with the first day discarded. A diagnostic target of ≥135/85 mmHg is recommended for all out-of-office measurements [38]. In the USA, the Eighth Joint National Committee (JNC 8) report updated the threshold for a diagnosis of hypertension in those aged 60 years and over, and those with diabetes or chronic kidney disease, but maintained previous recommendations on the use of clinic BP for routine diagnosis of hypertension [62]. More recent evidence reviews in America have recommended updating guidelines to include ABPM for all new diagnoses of hypertension and as a way of quickly diagnosing white coat or masked hypertension. Overall, the direction of travel in guidelines in Europe and North America is firmly towards greater use of out-of-office measurement of BP in the diagnosis of hypertension [49, 63].

### What is the evidence for treatment targets in hypertension?

Although it can be argued that the relationship between BP and cardiovascular risk is continuous, hypertension targets provide an essential management guide [64]. The Hypertension Optimal Treatment (HOT) trial, which compared three diastolic targets (≤90 mmHg, ≤85 mmHg and ≤80 mmHg), tried to provide definitive data and found no difference overall in the rate of cardiovascular events between targets [65]. Interestingly, there was a 51 % reduction in cardiovascular events between the ≤90 mmHg target group and ≤80 mmHg target group in diabetic patients, highlighting the need to consider different targets depending on the level of cardiovascular risk. Furthermore, post hoc analyses suggested that the lowest incidence of cardiovascular events was at 82.6 mmHg, whilst the lowest mortality rate was at 86.5 mmHg, and the small differences in achieved BP between groups have been criticised [65]. The use of diastolic targets has declined more recently with the evidence that systolic BP carries the greatest risk for heart disease and stroke, and this is reflected in most current guidelines [66, 67].

Over the last decade, work to establish accurate BP targets for treatment has been ongoing. A comprehensive meta-analysis by Law et al. reported a fall in cardiovascular disease (CVD) events by 25 %, a reduction in heart failure by about 25 % and in stroke by 33 % for every 10 mmHg drop in systolic BP and every 5 mmHg drop in diastolic BP without a lower threshold, at least to 110 mmHg systolic [68]. A recent individual patient data meta-analysis by the Blood Pressure Lowering Treatment Trialists’ Collaboration included over 50,000 patients and showed that lowering BP provided a similar relative risk reduction for all levels of cardiovascular risk [69]. However, the absolute risk reduction increased as the level of cardiovascular risk increased. For every 1,000 patients with >20 % cardiovascular risk, 38 CVD events could be prevented over 5 years, whereas for every 1,000 patients with a 6 % cardiovascular risk, 14 CVD events would be prevented. Therefore, patients at the highest risk stand to gain the most from having their BP lowered and arguably might benefit from lower targets [69]. Despite this, trials have failed to show benefit from intensive lowering of BP, particularly for older patients. The Valsartan in Elderly Isolated Systolic Hypertension (VALISH) study assessed a BP target of <140 mmHg against a more relaxed target of ≥140–150 mmHg in patients aged 70 years or older [70]. After 3 years, the number of composite cardiovascular events between the two target groups was not significant, although rather counterintuitively the authors concluded that the more stringent target was safe to initiate in older patients [70]. The Japanese Trial to Assess Optimal Systolic Blood Pressure in Elderly Hypertensive Patients (JATOS), which looked at the optimum systolic BP target in elderly hypertensive patients, also found no difference in cardiovascular disease or renal failure between patients with a systolic pressure ≤140 mmHg and those with a target ≤160 mmHg [71]. There was also no difference in mortality or adverse events between the groups. However, as the endpoints of both these trials were less frequent than expected, both were ultimately underpowered to answer whether tighter BP control was in fact superior to a more relaxed BP target.

The modelling work of Port et al. contradicts the epidemiological and trial evidence [64, 72] finding that the risk of CVD, stroke and death is stable below a cut-off at the 70th centile, with age- and sex-dependent increases in risk above this [73]. According to the thresholds proposed by Port et al., patients with a BP of 155 mmHg would not be treated. This suggestion has not been taken up internationally by guideline developers, other than for the elderly where the Hypertension in the Very Elderly Trial (HYVET) provided strong evidence for a similar treatment target [74], although Port's work has the potential merit of targeting treatment to younger high relative risk individuals.

Recent work examining the effectiveness of BP targets in a treated hypertension population of 398,419 has shown the presence of a J-shaped curve, where the highest rate of mortality came from lower and higher BP than the reference standard of 130–139/60–79 mmHg. The nadir for systolic and diastolic BP was 137/69 mmHg, but stratified analysis for patients with diabetes showed that the nadir was slightly lower at 131/69 mmHg, while patients ≥70 years of age had a nadir of 140/70 mmHg [75]. This supports the need for different targets for different patient groups, but does suggest that recent rowing back from lower (especially systolic) targets in uncomplicated hypertension is probably justified. A recent Cochrane review in 2012 assessed the benefit of pharmacotherapy in patients with mild, uncomplicated hypertension (140–149 mmHg systolic and/or 90–99 mmHg diastolic) [76]. For 7,080 participants, treatment with antihypertensive drugs compared to placebo did not lead to significant differences in the relative risk of total mortality, coronary heart disease, stroke or cardiovascular events, and withdrawals due to adverse events were increased by antihypertensive drugs [76]. This is reflected in the risk-based approach taken by NICE and the New Zealand guidelines in terms of only treating patients with stage 1 hypertension at higher risk and by JNC 8, albeit controversially in their relaxation of targets for older people [37, 62, 77].

However, the time taken to bring patients to the optimum systolic target can also impact on mortality outcomes. Xu et al. investigated the time to intensification of treatment and the time to follow-up for new medications [78]. Delays in hypertension treatment intensification of 1.4 months or more and delays in BP follow-up after treatment intensification of 2.7 months or more resulted in increased likelihood of an acute cardiovascular event or death by 1.12 and 1.18, respectively. However, a systolic target of 150 mmHg performed similarly to one of 140 mmHg, with the greatest risk of cardiovascular events or death seen at systolic intensification targets of 160 mmHg or more and a hazard ratio of at least 1.21 [78].

Earlier this year, Zanchetti et al. carried out a meta-analysis on 68 BP lowering trials to try and answer questions about the effectiveness of current thresholds [79]. They found that all-cause mortality, including stroke, cardiovascular, coronary heart disease and heart failure, were all significantly reduced by lowering systolic BP below 150 mmHg. With the exception of heart failure, outcomes could be further reduced by reducing systolic BP to 140 mmHg. Below this, only stroke was significantly reduced by decreasing systolic BP to 130 mmHg. Similarly, for diastolic BP, a significant reduction of cardiovascular outcomes could be seen at a cut-off of <90 mmHg, but only significant reductions in stroke could be seen for a target of <80 mmHg.

### Current target recommendations

NICE reviewed three systematic reviews and 27 prognostic studies to develop their current treatment threshold recommendations [37]. The cut-off points for ABPM were set lower than those for clinic BP. Evidence for these thresholds came largely from work comparing clinic BP and ABPM to cardiovascular outcomes [80, 81]. The Pressioni Arteriose Monitorate e Loro Associazioni (PAMELA) trial was a landmark study in this area and the authors predicted that a clinic BP of 140/90 mmHg was equivalent to a 24-hour ABPM of 125/80 mmHg or a daytime ABPM of 130/85 mmHg [81]. However, this work was based on prognostic thresholds and there is very little evidence for comparable treatment targets between ABPM and clinic BP. As discussed above, Head et al. analysed clinic BP and ABPM measurements from patients referred to hypertension clinics across Australia [34, 80]. The treatment target recommendations for ABPM and SMBP (Table 1) from the NICE guidelines are largely based on this work.

**Table 1:** Comparison of recommendations for diagnostic and treatment thresholds between NICE, ESH, JNC and CHEP guidelines

Organisation	Year	Diagnostic threshold	Treatment threshold
UK National Institute for Health and Care Excellence (NICE)	2011	OBPM diagnostic threshold: ≥140/90 mmHg (ABPM/HBPM diagnostic threshold: ≥135/85 mmHg)	All patients under 80 yrs: OBPM < 140/90 mmHg (ABPM/HBPM: <135/85 mmHg)
		OBPM Stage 2 Hypertension ≥160/100 mmHg (ABPM/HBPM: ≥150/95 mmHg)	Diabetes : OBPM <140/80 mmHg (or <130/80 mmHg if complications present)
			Older ≥80 yrs: OBPM <150/90 mmHg (ABPM/HBPM <145/85 mmHg)
European Society of Hypertension (ESH)	2013	OBPM: ≥140 and/or ≥90	All patients under 80 yrs OBPM <140/90 mmHg
		(ABPM Daytime (or awake): ≥135 and/or ≥85 mmHg, ABPM Night-time (or asleep): ≥120 and/or ≥70 mmHg, ABPM 24-h: ≥130 and/or ≥80 mmHg, HBPM: ≥135 and/or ≥85 mmHg)	Diabetes: OBPM <140/85 mmHg
			High Risk Patients: OBPM <130/80 mmHg
			Older ≥80 yrs: OBPM <150/90 mmHg
Joint National Committee on Prevention, Detection, Evaluation and Treatment of Blood Pressure (JNC 7)	2004	Stage 1 hypertension diagnosis should be confirmed within 2 months after initial elevated OBPM ≥140/90 mmHg	Non-diabetic patients: OBPM <140/90 mmHg
			Diabetic/CKD patients: OBPM <130/80 mmHg
		Stage 2 hypertension should be confirmed within 1 m	
		≥180/110 mm Hg evaluate and treat immediately	
		(ABPM Daytime (or awake): ≥135/85 mmHg, ABPM Night-time (or asleep): ≥120/75 mmHg)	
		Routine blood pressure measurements should be taken	
		− at least once every 2 years for adults with <120/80 mmHg	
		− every year for adults with 120-139/80-89 mmHg	
Joint National Committee on Prevention, Detection, Evaluation and Treatment of Blood Pressure (JNC 8)	2014	• Guidelines did not address diagnostic thresholds of hypertension.	Age <60 yrs: OBPM <140/90 mmHg
			Diabetes No CKD: OBPM <140/90 mmHg
		• The supplementary material recommends averaging 2–3 measurements at each visit to establish a diagnosis of hypertension.	CKD present with or without diabetes: OBPM <140/90 mmHg
			Older ≥60 years: OBPM <150/90 mmHg
		• Thresholds for pharmacological treatment were defined.	
		• HBPM and ABPM were not included.	
Canadian Hypertension Education Program (CHEP)	2015	ABPM Daytime (or awake): ≥135/85 mmHg	All ages <80 yrs: OBPM <140/90 mmHg
		ABPM 24-h: ≥130/80 mmHg	Diabetes: OBPM <130/80 mmHg
		HBPM diagnostic threshold: ≥135/85 mmHg)	Older ≥80 yrs: OBPM <150 mmHg
		OBPM diagnostic threshold:	
		≥140/90 mmHg averaged across two visits;	
		≥160/110 mmHg averaged across three visits;	
		or if ≥140/90 mmHg averaged across five visits	

*Abbreviations:* *OBPM* office blood pressure measurement, *ABPM* ambulatory blood pressure measurement, *HBPM* home blood pressure measurement, *CKD* chronic kidney disease

NICE suggests an ABPM treatment target of <135/85 mmHg for patients below 80 years of age and an ABPM target of <145/85 mmHg for those patients over 80 years [37]. Currently, they are the only guidelines to specify a common treatment and management threshold for ABPM with the same thresholds assumed for home BP monitoring.

The ESH 2013 guidelines suggest a target of <140 mmHg for all patients under 80 years of age and a diagnostic target of <90 mmHg. Diabetic patients should be treated to a diastolic target of <85 mmHg. Patients over 80 years with a systolic BP ≥160 mmHg should be treated to a target between 140 and 150 mmHg according to the evidence [5].

The recent JNC 8 guideline recommended treating those between 18 and 60 years old to a target of 140/90 mmHg [62]. However, for those patients above 60 years, they recommended a systolic treatment target of <150 mmHg. This caused some controversy, but the committee argued that evidence from the trials they had considered found little benefit of tighter control leading to better outcomes [62]. However, many countered that these recommendations were based on a very small number of trials which passed the rigorous screening process, excluding evidence from meta-analysis, and meaning that this recommendation was almost entirely based on expert opinion [82].

## Conclusion and future directions

Due to its diagnostic accuracy, ABPM seems set to assume greater prominence for the new diagnosis of hypertension – it is already in place in the NICE and arguably the ESH guidelines [5, 37] and there are strong recommendations for the USA and Canada to follow suit [49, 63]. Less developed countries may reasonably continue with clinic measurement. It is likely that self-monitoring will also feature strongly and this is seen in the latest Japanese guidance [83]. There is strong evidence for the use of out-of-office measurements to diagnose and manage patients with white coat and masked hypertension, with important implications for the appropriate targeting of treatment.

Outside of primary care, other health professionals, such as pharmacists, dentists and optometrists, are starting to have more of a role in monitoring BP. There will need to be clear pathways set up between these organisations and primary care providers in order to follow-up cases of high BP readings [55]. Self-monitoring is also starting to be used for self-screening [55] and we may see a role for this in patients identified as having high-normal BP. Robust diagnostic guidelines will be needed to advise both physicians and non-physicians how to obtain accurate out-of-office measurements. The use of smartwatches for health tracking may drive greater telemonitoring, but for the moment cuff-based measurements limit their use in hypertension.

Measurement of blood pressure is well captured by the guidelines, but what is less clear is how to ensure adequate population screening. Further studies should seek to establish optimum rescreening intervals, alternative ways to gain full population coverage and methods to identify those at greatest risk.

Many trials have shown that controlling blood pressure is an important way of reducing heart disease and stroke [68, 74, 84, 85]. Blood pressure targets are an essential clinical tool for guiding control of blood pressure. However, questions about the optimum targets remain, particularly for patient subpopulations, such as the elderly, those with high cardiovascular risk and certain ethnic groups [86]. Studies in this area should focus on the optimum BP threshold to initiate treatment, but also perhaps when to reduce treatment, particularly in the context of polypharmacy [87].

Overall, the mainstay of hypertension diagnosis and monitoring is likely to remain systematically identifying and then treating patients to fairly conventional targets, with use of out-of-office monitoring for both diagnosis and management. Ensuring physicians do not succumb to clinical inertia is probably as important as the actual methods and targets used [88].

## Abbreviations

AAMI: Association for the Advancement of Medical Instrumentation
ABPM: Ambulatory blood pressure monitoring
BP: Blood pressure
CHEP: Canadian Hypertension Education Program
CVD: Cardiovascular disease
ESH: European Society of Hypertension
HOT: Hypertension Optimal Treatment
HYVET: Hypertension in the Very Elderly Trial
IDACO: International Database of Ambulatory Blood Pressure in relation to Cardiovascular Outcome
JATOS: Japanese Trial to Assess Optimal Systolic Blood Pressure in Elderly Hypertensive Patients
JNC 8: Eighth Joint National Committee
NICE: National Institute for Health and Care Excellence
PAMELA: Pressioni Arteriose Monitorate e Loro Associazioni
RCT: Randomised controlled trial
SMBP: Self-measured blood pressure
VALISH: Valsartan in Elderly Isolated Systolic Hypertension
